# M-current induced Bogdanov–Takens bifurcation and switching of neuron excitability class

**DOI:** 10.1186/s13408-021-00103-5

**Published:** 2021-02-15

**Authors:** Isam Al-Darabsah, Sue Ann Campbell

**Affiliations:** Department of Applied Mathematics and Centre for Theoretical Neuroscience, University of Waterloo, N2L 3G1 Waterloo, ON Canada

**Keywords:** Conductance-based models, Bogdanov–Takens bifurcation, Neuronal excitability, M-current

## Abstract

In this work, we consider a general conductance-based neuron model with the inclusion of the acetycholine sensitive, M-current. We study bifurcations in the parameter space consisting of the applied current $I_{app}$, the maximal conductance of the M-current $g_{M}$ and the conductance of the leak current $g_{L}$. We give precise conditions for the model that ensure the existence of a Bogdanov–Takens (BT) point and show that such a point can occur by varying $I_{app}$ and $g_{M}$. We discuss the case when the BT point becomes a Bogdanov–Takens–cusp (BTC) point and show that such a point can occur in the three-dimensional parameter space. The results of the bifurcation analysis are applied to different neuronal models and are verified and supplemented by numerical bifurcation diagrams generated using the package MATCONT. We conclude that there is a transition in the neuronal excitability type organised by the BT point and the neuron switches from Class-I to Class-II as conductance of the M-current increases.

## Introduction

Neuromodulators are chemicals released by neurons that can alter the behaviour of individual neurons and large populations of neurons. Examples include dopamine, seratonin and acetylcholine. These chemicals occur widely in the brain and can affect many types of neurons. The effect of neuromodulators ranges from altering the membrane properties of individual neurons to altering synaptic transmission.

The M-current is a voltage-dependent, non-inactivating potassium current, which has been shown to occur in many neural types including excitatory neurons in the cortex [1] and inhibitory neurons in the hippocampus [2]. Its name arises from the fact that this current is down-regulated by the presence of the neuromodulator acetylcholine through its action on the muscarinic receptor. At the simplest level, this current reduces firing activity since it is a potassium current [2, 3]. However, this current has been implicated in many aspects of both individual cell and network activity.

Before reviewing the literature on the M-current, we first recall some terminology. Neurons and neural models are often classified by their membrane excitability class. As described by Hodgkin [4], neurons with the Class-I excitability have a continuous frequency-current ($\mathrm{F}/\mathrm{I}$) curve because they begin repetitive firing with zero frequency from the resting state. On the other hand, the frequency-current curve of Class-II neurons is discontinuous because they start firing with non-zero frequency from the resting state [5]. The phase resetting curve (PRC) describes the effect of stimulation on the phase of an oscillator as a function of the phase at which the stimulus is delivered. At phases where the PRC is positive the phase is advanced, meaning the period of the oscillator is increased by the perturbation. At phases where the PRC is negative the phase is delayed, corresponding to a decrease in the period of the oscillator [6, 7]. As introduced by Hansel [8], a Type-I PRC is one where an excitatory stimulus produces only phase advances, while in a Type-II PRC either phase advance or phase delay can occur, depending on the phase of the stimulus. For two oscillators with reciprocal excitatory coupling, a Type-I PRC means the coupling cannot synchronise the oscillators, while a Type-I PRC means that the coupling can synchronise the oscillators. For inhibitory coupling, the opposite occurs [6, 7]. Another important classification of neurons is whether or not they exhibit subthreshold oscillations. Neurons that do exhibit subthreshold oscillations are called resonators, while neurons that do not are called integrators.

At the single cell level, the M-current has been shown to affect the neuronal excitability [1, 9] and resonant properties [10–12]. For example, in [1], the authors recorded from layer II/III pyramidal neurons and determined PRCs. Stiefel et al. [1] found that down-regulation of slow voltage-dependent potassium currents such as the M-current can switch the PRC from Type-II to Type-I, thus changing the expected synchronisation of pairs of coupled neurons. In a follow-up paper [1], they showed for that the M-current could produce the same effect in several different neural models. The work of [13] showed that these differences in PRC type due to M-current modulation translate into differences in synchronisation properties in networks of model neurons. The experimental work of [11, 12] showed that increased membrane conductance (shunting) could switch a hippocampal pyramidal neuron from an integrator to a resonator. Using a simple model, they attributed this change to the combined effect of shunting (modelled as a leak current) and the M-current. Interpreting the shunt as representing the effect of background synaptic on a neuron, Prescott et al. [12] concluded that neurons that present as integrators in vitro may act as resonators in vivo. At the network level the M-current has also been implicated in the organisation of rhythms in striatal microcircuits. In [14], the authors studied an inhibitory neuron model with M-current under forcing from gamma pulses and a sinusoidal current of theta frequency. They found that the M-current expands the phase-locking frequency range of the network, counteracts the slow theta forcing and admits bistability in some parameter range. In [15], the effects of the M-correct on *β* oscillations was studied.

In all the studies cited above, the effect of acetylcholine, through the M-current, was explored in models for specific cells. While this is important for understanding the behaviour of specific cells and brain networks, it can be difficult to extract the essential effects of the M-current from its interplay with other specific currents in the models. Here we take a different approach and consider the effect of the M-current in a general conductance-based model. We study the bifurcations of the model in the parameter space of two parameters common to any conductance-based model with an M-current: the applied current and the maximal conductance of the M-current. We derive necessary and sufficient conditions for the existence of two codimension two bifurcations of the resting equilibrium point: the Bogdanov–Takens (BT) bifurcation and the cusp (CP) bifurcation.

The Bogdanov–Takens (BT) bifurcation is associated with an equilibrium point that has a zero eigenvalue with algebraic multiplicity two and geometric multiplicity one. The cusp bifurcation occurs when three equilibrium points coalesce into one, and can be thought of as the simultaneous occurrence of two fold bifurcations. When an equilibrium point simultaneously undergoes BT and cusp bifurcations, a Bogdanov–Takens–cusp (BTC) occurs, which is a codimension three bifurcation. We show that variation of a third parameter, the leak conductance, can lead to a BTC bifurcation point.

In the literature, there are many instances where the presence of the BT, cusp and to a lesser extent the BTC, bifurcations has been shown to occur in particular conductance-based models. For example, the presence of BT and cusp bifurcations [16] and BTC bifurcation [17] has been shown in the Hodgkin–Huxley model. In [18], the author showed the existence of BT and cusp bifurcations in Morris–Lecar model [19]. While in [7] the BT and cusp bifurcations were shown in the Wang–Buzsáki interneuron model [20]. The majority of these studies used numerical bifurcation analysis to show that these bifurcations occur as particular parameters are varied with all other parameters fixed at some specific, biologically relevant values. The prevalence of these codimension two bifurcations in particular studies would seem to indicate that these bifurcations are associated with some underlying structure in conductance-based models in general. Indeed, two recent papers give support to this hypothesis. The authors in [21] considered a general conductance-based neuron model and studied the existence of the BTC point in the parameter space of the applied current, leak conductance and capacitance. In [22], the authors give general conditions for the existence of the BT bifurcation in any conductance-based model. Our work builds on these latter two papers and extends them to the situation where an M-current is present in the model.

To understand the implications of the codimension two bifurcations, we related them to the neural behaviours described above. The resonance property of neural models is quite simply related to the bifurcation that causes the loss of stability of the resting state when the applied current is increased. If this bifurcation is a Hopf bifurcation the model is a resonator, otherwise it is an integrator. As pointed out by Izhikevich, a Bogdanov–Takens bifurcation can switch the resonator type of a neuron [5]. Class I/II excitability was first linked to bifurcations in neural models by Rinzel and Ermentrout [23]. Rinzel and Ermentrout showed that neuronal models where the onset of repetitive firing occurs via a saddle-node bifurcation on an invariant circle are Class-I, while models where the onset occurs via a subcritical Andronov–Hopf bifurcations are Class-II. This link can be extended to other types of bifurcations by studying the associated $\mathrm{F}/\mathrm{I}$ curves. The excitability class of individual neurons has been linked to the synchronisation properties of the neuron in a network through the phase resetting curve (PRC). In particular, it has been shown in certain circumstances that Class-I neurons have Type-I PRCs [24]. No conclusive link between Class-II neurons and a particular PRC type was found in that paper. More recently, Izhikevich has made a subtly different classification of excitability based on ramped current inputs as opposed to step current inputs. Izhikevich defines Class I/II excitability based on the bifurcation that causes the loss of stability of the resting state when the current is increased. Further, Izhikevich defines Class I/II spiking by the bifurcation that destroys the stable oscillations as the current is decreased [5]. A focus for this paper will be on how the presence of a BT point is linked to the emergence of a Hopf bifurcation and thus could be associated with a change of oscillation class for a conductance-based neural model.

The paper is organised as follows. In the next section, we provide a general conductance-based neuron model with the inclusion of the *M*-current and study the existence of the steady-state solutions. In Sects. 3 and 4, we give a complete characterisation of the BT bifurcation, provide a condition for the cusp bifurcation and discuss the existence of Bogdanov–Takens–cusp (BTC) bifurcation. In Sect. 5, we consider three example models and show that all three models exhibit the BT, CP and BTC bifurcation points. We construct bifurcation diagrams using MATCONT to explain possible behaviour of each example and use the numerical solution of each model to construct the frequency-current curves. In Sect. 6, we use numerical simulations to study the influence of varying of $g_{M}$ on the neurons synchronisation in two coupled neurons model with synaptic coupling. In Sect. 7, we discuss our results.

## General model

In non-dimensional variables, a general conductance-based neuron model with the inclusion of the *M*-current can be written as follows:

1 $$ \begin{gathered} {C_{m}}\frac{{dV}}{{dt}} = {I_{app}} - {g_{L}} ( {V - {V_{L}}} ) - {g_{M}}w ( {V - {V_{K}}} ) + {I_{ion}}(V,a), \\ \frac{{dw}}{{dt}} = \frac{1}{{{r}(V)}} \bigl( {{w_{\infty }}(V) - w} \bigr), \\ \frac{{d{a}}}{{dt}} ={\tau }^{-1}(V) \bigl( {{a_{\infty }}(V) - {a}} \bigr), \end{gathered} $$

where $a = { ({a_{3}},\ldots,{a_{N}} )^{T}}$,

$$ {a_{\infty }}(V) = { \bigl({a_{3,\infty }}(V),\ldots,{a_{N,\infty }}(V) \bigr)^{T}},\quad\quad \tau ^{-1} (V) = \operatorname{diag} \biggl(\frac{1}{{\tau _{3}}(V)},\ldots,\frac{1}{{\tau _{N}}(V)} \biggr) $$

and

$$ {I_{ion}}(V,a) = \sum_{i = 3}^{N} {{g_{i}} ( {{V_{i}} - V} )\prod _{j \in {\phi _{i}}} {a_{j}^{{p_{j}}}} }, $$

where ${I_{app}}$ is the applied current and $\phi _{i}$ is the set of indexes that represents the identities of the gating variables present in a given ionic current. In the rest of the manuscript, we assume that all conductances $g_{j}$ are positive, and the steady state activations $w_{\infty }$ and $a_{j,\infty }$, $j=3,\ldots,N$, are non-negative bounded functions ($0\le f(V)\le 1$), monotonic $C^{3}(\mathbb{R},\mathbb{R})$ and become sufficiently flat in the limits $V\to \pm \infty $.

### Equilibria

By applying the scaling $t\to \frac{t}{C_{m}}$, system (1) can be written as

2 $$\begin{aligned}& \frac{{dV}}{{dt}} = {I_{app}} - {g_{L}}(V-{V_{L}}) - {g_{M}}w ( {V - V_{K}} ) + {I_{ion}}(V,a):=f_{1}(V,w,a), \\& \frac{{dw}}{{dt}} = \frac{C_{M}}{{r(V)}} \bigl( {{w_{\infty }}(V) - w} \bigr):=f_{2}(V,w), \\& \frac{{da}}{{dt}} = C_{M} {\tau ^{-1} (V)} \bigl( {{a_{\infty }}(V) - a} \bigr):=f_{3}(V,a), \end{aligned}$$

where $f_{3}(V,a)= (f_{33}(V,a_{3}),\ldots, f_{3N}(V,a_{N}) )^{T}$. Assume that (2) has an equilibrium point $E^{{*}}=(V^{*},w^{*},a_{0}^{*})$. From the equations above it follows that

$$ {w_{\infty }}\bigl({V^{*}}\bigr) = {w^{*}}\quad \text{and}\quad {a_{\infty }}\bigl({V^{*}}\bigr) = {a^{*}}, $$

where $V^{*}$ satisfies

3 $$\begin{aligned} I_{\infty }\bigl(V^{*}\bigr)=0. \end{aligned}$$

Here, $I_{\infty }$ is the steady-state $I-V$ curve [5, 23] defined by

4 $$ I_{\infty }(V)={I_{app}} - {g_{L}}(V-{V_{L}}) - {g_{M}}w_{\infty }(V) ( {V - V_{K}} ) + {I_{ion,\infty }}(V), $$

where ${I_{ion,\infty }}(V) = {I_{ion}}(V,{a_{\infty }}(V))$ is the stationary ionic current. Notice that (3) can be written as

$$ {I_{app}} = {g_{L}}\bigl({V^{*}}-V_{L} \bigr)+{g_{M}} {w_{\infty }}\bigl(V^{*}\bigr) \bigl( {{V^{*}} - V_{K}} \bigr) - {I_{ion}} \bigl(V^{*},{a_{\infty }}\bigl({V^{*}}\bigr)\bigr) :=U \bigl(V^{*}\bigr). $$

Now, we write $U(V^{*})$ in the form

$$ U\bigl(V^{*}\bigr)= \bigl( {{g_{L}} + {g_{M}} {w_{\infty }}\bigl(V^{*}\bigr) + {h_{2}} \bigl(V^{*}\bigr)} \bigr)V^{*}- \bigl( { {g_{M}} {m_{\infty }}\bigl(V^{*}\bigr) + {h_{1}} \bigl(V^{*}\bigr)} \bigr)- {g_{L}} {V_{L}}, $$

where $h_{1}$ and $h_{2}$ are polynomials in the variables $a_{j,\infty }(V)$, and hence

$$ \lim_{V^{*} \to \pm \infty } U\bigl(V^{*}\bigr) = \pm \infty $$

because all maximal conductances and activation variables are positive and bounded. Thus, equation (3) has at least one solution.

## Bogdanov–Takens bifurcation

In the following we discuss Bogdanov–Takens point (BT point) of codimension two in $(I_{app}, g_{M})$-plane, when all other parameters in the model are fixed.

Assume that $V^{*}$ is a solution of (3), then there exist parameters $(I_{app}^{*},g^{*}_{M})$ such that

5 $$ {I_{app}^{*}} = {g_{L}} \bigl({V^{*}}-V_{L}\bigr) + {g^{*}_{M}} {w_{\infty }}\bigl({V^{*}}\bigr) \bigl( {{V^{*}} - V_{K}} \bigr) - {I_{ion}}\bigl(V^{*},{a_{\infty }} \bigl({V^{*}}\bigr)\bigr). $$

It is well known [25–27] that the equilibrium point $V^{*}$ is a BT point if the zero eigenvalue has algebraic multiplicity two and geometric multiplicity one. Using an approach similar to [21, 22], we obtain the following.

### Theorem 3.1

*Let* $V^{*}$ *be a solution of* (3) *at* $(I_{app}^{*},g^{*}_{M})$ *and define*

$$ \partial _{a}^{{f_{1}}} = { \biggl( { \frac{{\partial {f_{1}}}}{{\partial {a_{3}}}} ,\ldots, \frac{{\partial {f_{1}}}}{{\partial {a_{N}}}}} \biggr)^{T}}, \quad\quad \partial _{V}^{{f_{3}}} = { \biggl( { \frac{{\partial {f_{33}}}}{{\partial V}} ,\ldots, \frac{{\partial {f_{3N}}}}{{\partial V}}} \biggr)^{T}}. $$

*Assume*

6 $$\begin{aligned}& { {\frac{d}{{dV}}{I_{\infty }}(V)} \bigg| _{{V^{*}}}} = 0, \end{aligned}$$

7 $$\begin{aligned}& 1 +\frac{r^{2}}{C_{M}^{2}}{ { \frac{{\partial {f_{1}}}}{{\partial w}}} \bigg| _{{E^{*}}}} { { \frac{{\partial {f_{2}}}}{{\partial V}}} \bigg| _{{E^{*}}}} + \frac{1}{C_{M}^{2}} \bigl( {{{ {\partial _{a}^{f_{1}^{T}}} \big| }_{{E^{*}}}}} \bigr){\tau ^{2}} \bigl( {{{ { \partial _{V}^{{f_{3}}}} \big| }_{{E^{*}}}}} \bigr) = 0. \end{aligned}$$

*Then* $E^{*}$ *is an ordinary BT point of codimension two*.

### Proof

Let $F=(f_{1},f_{2},f_{3})^{T}$. Then the Jacobian of (2) is

$$\begin{array}{r} DF(V,w,a)=\left(\begin{array}{ccc} \frac{\partial f_{1}}{\partial V} & \frac{\partial f_{1}}{\partial w} & \partial_{a}^{f_{1}^{T}}\\ \frac{\partial f_{2}}{\partial V} & -C_{M}r^{-1} & 0\\ \partial_{V}^{f_{3}} & 0 & -C_{M}\tau^{-1} \end{array}\right), \end{array}$$

where $r^{-1}=\frac{1}{r(V)}$, $\tau ^{-1} = \operatorname{diag} (\frac{1}{{\tau _{3}}(V)},\ldots, \frac{1}{{\tau _{N}}(V)} )$.

When ${B_{1}} \in {\mathbb{R}^{n \times n}}$, ${B_{2}} \in {\mathbb{R}^{n \times m}}$, ${B_{3}} \in {\mathbb{R}^{m \times n}}$, ${B_{4}} \in { \mathbb{R}^{m \times m}}$, we have (see [28])

$$\det\left(\begin{array}{cc} B_{1} & B_{2}\\ B_{3} & B_{4} \end{array}\right)=(\det B_{4})\det\left(B_{1}-B_{2}B_{4}^{-1}B_{3}\right).$$

Let $A=DF(V^{*},m^{*},a^{*})$. Then, by taking ${B_{1}} = ( {\frac{{\partial {f_{1}}}}{{\partial V}}} - \lambda )$, ${B_{2}} = ( {\frac{{\partial {f_{1}}}}{{\partial w}}}\ \ {\partial {{_{a}^{{f_{1}}}}^{T}}} )$, ${B_{3}} = ( {\frac{{\partial {f_{2}}}}{{\partial V}}}\ \ {\partial _{V}^{{f_{3}}}} )^{T}$ and ${B_{4}} = \operatorname{diag} ( { -C_{M} {r^{ - 1}}-\lambda , -C_{M} {\tau ^{ - 1}}-\lambda I} )$, we have

$$ \det (A-\lambda I):=\Delta (\lambda )=\Delta _{1}(\lambda )\Delta _{2}( \lambda ), $$

where

$$ {\Delta _{1}}(\lambda ) = {( - 1)^{N - 1}} \bigl( {\lambda + C_{M}{r^{ - 1}}} \bigr)\prod_{j = 3}^{N} { \bigl( {\lambda +C_{M} \tau _{j}^{ - 1}} \bigr)} $$

and

$$ {\Delta _{2}}(\lambda ) = \frac{{\partial {f_{1}}}}{{\partial V}} - \lambda + { \bigl( { \lambda +C_{M} {r^{ - 1}}} \bigr)^{ - 1}} \frac{{\partial {f_{1}}}}{{\partial w}} \frac{{\partial {f_{2}}}}{{\partial V}} + \partial _{a}^{f_{1}^{T}}{ \bigl( {\lambda I +C_{M} {\tau ^{ - 1}}} \bigr)^{ - 1}} \partial _{V}^{{f_{3}}}. $$

Consequently, we have

$$ \Delta (0) = {\Delta _{1}}(0){\Delta _{2}}(0) = {\Delta _{1}}(0) \biggl( {\frac{{\partial {f_{1}}}}{{\partial V}} +C_{M} r \frac{{\partial {f_{1}}}}{{\partial w}} \frac{{\partial {f_{2}}}}{{\partial V}} +C_{M} \partial _{a}^{f_{1}^{T}} \tau \partial _{V}^{{f_{3}}}} \biggr). $$

Notice that

8 $$\begin{aligned} { {\frac{{\partial {f_{2}}}}{{\partial V}}} \bigg| _{{E^{*}}}} = C_{M}{r^{ - 1}}\bigl(V^{*}\bigr) \biggl( {{{ { \frac{d}{{dV}}{w_{\infty }}(V)} \bigg| }_{{V^{*}}}}} \biggr),\qquad { { \partial _{V}^{{f_{3}}}} \big| _{{E^{*}}}} =C_{M} { \tau ^{ - 1}}\bigl(V^{*}\bigr){ {\partial _{V}^{{a_{\infty }}}} \big| _{{V^{*}}}}. \end{aligned}$$

Thus, at $E^{*}$, we have that the equation

9 $$\begin{aligned} \frac{{\partial {f_{1}}}}{{\partial V}} +\frac{r}{C_{M}} \frac{{\partial {f_{1}}}}{{\partial w}} \frac{{\partial {f_{2}}}}{{\partial V}} + \frac{1}{C_{M}} \partial {_{a}^{{f_{1}^{T}}}} \tau \partial _{V}^{{f_{3}}} = 0 \end{aligned}$$

is equivalent to ${ {\frac{d}{{dV}}{I_{\infty }}(V)} | _{{V^{*}}}} = 0$. Thus, $\Delta (0)=0$ when (6) holds.

It easy to check that

$$ \begin{aligned} \Delta '(0) &= {\Delta _{1}}(0)\Delta ' _{2}(0) + \Delta ' _{1}(0){ \Delta _{2}}(0) \\ &= - {\Delta _{1}}(0) \biggl( {1 + \frac{r^{2}}{C_{M}^{2}} \frac{{\partial {f_{1}}}}{{\partial w}} \frac{{\partial {f_{2}}}}{{\partial V}} + \frac{1}{C_{M}^{2}} \partial _{a}^{f_{1}^{T}}{\tau ^{2}}\partial _{V}^{{f_{3}}}} \biggr) \\ & \quad{} + \Delta ' _{1}(0) \biggl( { \frac{{\partial {f_{1}}}}{{\partial V}} +C_{M} r \frac{{\partial {f_{1}}}}{{\partial w}} \frac{{\partial {f_{2}}}}{{\partial V}} +C_{M} \partial _{a}^{f_{1}^{T}} \tau \partial _{V}^{{f_{3}}}} \biggr). \end{aligned} $$

Thus, at $E^{*}$, $\Delta '(0)=0$ when (6) and (7) hold. Hence, $\lambda =0$ is a double root.

Now, we show that a Jordan block arises when $\lambda =0$ is a double multiplicity root. In other words, when (6) and (7) hold, we demand the existence of four generalised eigenvectors $q_{0}$, $q_{1}$, $p_{0}$, $p_{1}$ of *A* such that

$$ Aq_{0}=0,\quad\quad Aq_{1}=q_{0},\quad\quad A^{T}p_{1}=0,\quad\quad A^{T}p_{0}=p_{1}. $$

Let $q_{i}=(q_{i1},\ldots,q_{iN})^{T}$ and $p_{i}=(p_{i1},\ldots,p_{iN})^{T}$ for $i\in \{0,1\}$. Then we obtain from $Aq_{0}=0$ the following equations:

10 $$\begin{aligned}& {q_{01}}\frac{{\partial {f_{1}}}}{{\partial V}} + {q_{02}} \frac{{\partial {f_{1}}}}{{\partial w}} + \partial {_{a}^{{f_{1}^{T}}}} { ( {{q_{03}} , \ldots,{q_{0N}}} )^{T}} = 0, \end{aligned}$$

11 $$\begin{aligned}& {q_{01}}\frac{{\partial {f_{2}}}}{{\partial V}} - {q_{02}}C_{M}{r^{ - 1}} = 0, \end{aligned}$$

12 $$\begin{aligned}& {q_{01}}\frac{{\partial {f_{3j}}}}{{\partial V}} - \tau _{j}^{ - 1}{q_{0j}} = 0,\quad j = 3,\ldots,N. \end{aligned}$$

From (11) and (12), we have

$$ {q_{02}} = {q_{01}}\frac{r}{C_{M}} \frac{{\partial {f_{2}}}}{{\partial V}} \quad \text{and}\quad {q_{0j}} = {q_{01}}\frac{\tau _{j}}{C_{M}} \frac{{\partial {f_{3j}}}}{{\partial V}},\quad j = 3,\ldots,N, $$

respectively. Hence,

$$q_{0}=q_{01}\left(\begin{array}{c} 1\\ \frac{r}{C_{M}}\frac{\partial f_{2}}{\partial V}\\ \frac{\tau }{C_{M}}\partial_{V}^{f_{3}} \end{array}\right),$$

and it follows from (10) that

13 $$ {q_{01}} \biggl(\frac{{\partial {f_{1}}}}{{\partial V}} + \frac{r}{C_{M}} \frac{{\partial {f_{1}}}}{{\partial w}} \frac{{\partial {f_{2}}}}{{\partial V}} +\frac{1}{C_{M}} \partial {_{a}^{{f_{1}^{T}}}} \tau \partial _{V}^{{f_{3}}} \biggr) = 0. $$

Similarly, from $Aq_{0}=q_{1}$, $A^{T}p_{1}=0$ and $A^{T}p_{1}=p_{0}$, we have

$$\begin{array}{c} q_{1}=\left(\begin{array}{c} q_{11}\\ (q_{11}-q_{01}\frac{r}{C_{M}})\frac{r}{C_{M}}\frac{\partial f_{2}}{\partial V}\\ (q_{11}I_{N-3}-q_{01}\frac{\tau }{C_{M}})\frac{\tau }{C_{M}}\partial_{V}^{f_{3}} \end{array}\right),\;\;p_{1}=p_{11}\left(\begin{array}{c} 1\\ \frac{r}{C_{M}}\frac{\partial f_{1}}{\partial w}\\ \frac{\tau }{C_{M}}\partial_{a}^{f_{1}} \end{array}\right),\\ p_{0}=\left(\begin{array}{c} p_{01}\\ (p_{01}-p_{11}\frac{r}{C_{M}})\frac{r}{C_{M}}\frac{\partial f_{1}}{\partial w}\\ (p_{01}I_{N-3}-p_{11}\frac{\tau }{C_{M}})\frac{\tau }{C_{M}}\partial_{a}^{f_{1}} \end{array}\right), \end{array}$$

where $I_{N-3}$ is the identity matrix of size $N-3$, and

14 $$\begin{aligned} &{q_{11}} \biggl( {\frac{{\partial {f_{1}}}}{{\partial V}} + \frac{r}{C_{M}} \frac{{\partial {f_{1}}}}{{\partial w}} \frac{{\partial {f_{2}}}}{{\partial V}} +\frac{1}{C_{M}} \partial _{a}^{f_{1}^{T}} \tau \partial _{V}^{{f_{3}}}} \biggr) \\ &\quad{} - {q_{01}} \biggl( {1 + \frac{r^{2}}{C_{M}^{2}} \frac{{\partial {f_{1}}}}{{\partial w}} \frac{{\partial {f_{2}}}}{{\partial V}} + \frac{1}{C_{M}^{2}} \partial _{a}^{f_{1}^{T}}{\tau ^{2}}\partial _{V}^{{f_{3}}}} \biggr) = 0, \end{aligned}$$

15 $$\begin{aligned} & {p_{11}} \biggl( {\frac{{\partial {f_{1}}}}{{\partial V}} + \frac{r}{C_{M}} \frac{{\partial {f_{1}}}}{{\partial w}} \frac{{\partial {f_{2}}}}{{\partial V}} +\frac{1}{C_{M}} \partial _{V}^{f_{3}^{T}} \tau \partial _{a}^{{f_{1}}}} \biggr) = 0, \end{aligned}$$

16 $$\begin{aligned} & {p_{01}} \biggl( {\frac{{\partial {f_{1}}}}{{\partial V}} + \frac{r}{C_{M}} \frac{{\partial {f_{1}}}}{{\partial w}} \frac{{\partial {f_{2}}}}{{\partial V}} + \frac{1}{C_{M}}\partial _{V}^{f_{3}^{T}} \tau \partial _{a}^{{f_{1}}}} \biggr) \\ &\quad{} - {p_{11}} \biggl( {1 + \frac{r^{2}}{C_{M}^{2}} \frac{{\partial {f_{1}}}}{{\partial w}} \frac{{\partial {f_{2}}}}{{\partial V}} +\frac{1}{C_{M}^{2}} \partial _{V}^{f_{3}^{T}}{\tau ^{2}}\partial _{a}^{{f_{1}}}} \biggr) =0. \end{aligned}$$

As the generalised eigenvectors must be non-zero, we let $q_{01}$ and $p_{11}$ be non-zero arbitrary constants. Thus, when (6) and (7) hold, equations (13)–(16) hold. Thus, four generalised eigenvectors exist. Hence, $V^{*}$ is an ordinary Bogdanov–Takens point. □

### Remark 3.1

With the additional condition

$$ p_{i}^{T}{q_{j}} = \textstyle\begin{cases} 1&\text{if }i = j, \\ 0&\text{if }i \ne j, \end{cases} $$

we can guarantee the uniqueness of the generalised eigenvectors $q_{0}$, $q_{1}$, $p_{0}$, $p_{1}$ of *A*.

When $V^{*}$ is a BT point, system (2) has a two-dimensional centre manifold, with normal form given by (see, e.g. [25–27, 29, 30])

17 $$\begin{aligned} \begin{gathered} \frac{{d{\xi _{0}}}}{{dt}} = {\xi _{1}}, \\ \frac{{d{\xi _{1}}}}{{dt}} = {\alpha _{2}}\xi _{0}^{2} + {\beta _{2}} { \xi _{0}} {\xi _{1}} + O \bigl( {{{ \bigl\Vert { ( {{\xi _{0}},{\xi _{1}}} )} \bigr\Vert }^{3}}} \bigr), \end{gathered} \end{aligned}$$

where

18 $$\begin{aligned} \begin{gathered} {\alpha _{2}} = \frac{1}{2}p_{1}^{T}G({q_{0}},{q_{0}}), \\ {\beta _{2}} = p_{1}^{T}G({q_{0}},{q_{1}}) - p_{1}^{T}{h_{20}}, \end{gathered} \end{aligned}$$

where $h_{20}$ is the solution of the equation

19 $$ A{h_{20}} = 2{\alpha _{2}} {q_{1}} - G({q_{0}},{q_{0}}), $$

and the function *G* is defined as

$$G(z_{1},z_{2}):=\left(\begin{array}{c} z_{1}^{T}D^{2}f_{1}(V^{*})z_{2}\\ z_{1}^{T}D^{2}f_{2}(V^{*})z_{2}\\ z_{1}^{T}D^{2}f_{3}(V^{*})z_{2} \end{array}\right).$$

Here, ${D^{2}}f={ ( { \frac{{{\partial ^{2}}f}}{{\partial {x_{i}}\partial {x_{j}}}}} )_{1 \le i,j \le N}}$ is the Hessian matrix of a quadratic form at $V^{*}$.

### Bogdanov–Takens–cusp bifurcation

The steady state $V^{*}$ becomes a degenerate Bogdanov–Takens point (or “Bogdanov–Takens–cusp point”-BTC point) when a BT point combines with a cusp. A BTC occurs if either: Case 1: $\alpha _{2}=0$ and $\beta _{2}\ne 0$; or Case 2: $\alpha _{2}\ne 0$ and $\beta _{2}= 0$, see, e.g. [26]. Considering Case 1 and applying an approach similar to [21] with the results of [26], we have the following.

#### Theorem 3.2

*Assume that* $V^{*}$ *is an ordinary BT point*. *If*

20 $$ { {\frac{d^{2}}{{dV^{2}}}{I_{\infty }}(V)} \bigg| _{{V^{*}}}} = 0, $$

*then* $\alpha _{2}=0$ *and* $\beta _{2}\ne 0$, *that is*, $V^{*}$ *becomes a cusp*.

#### Proof

From $f_{m}$ and $f_{a}$, we have

$$ \frac{{\partial {f_{2}}}}{{\partial w}} = \frac{{ - C_{M}}}{r} \quad \Rightarrow \quad \frac{{{\partial ^{2}}{f_{1}}}}{{\partial {w^{2}}}} = 0 \quad \text{and}\quad \partial _{a}^{{f_{3}}} = - C_{M}{\tau ^{ - 1}} \quad \Rightarrow \quad \partial _{aa}^{{f_{3}}} = 0. $$

Hence, the components of *G* are

$$\begin{aligned}& \begin{aligned} \frac{1}{{q_{01}^{2}}}q_{0}^{T}{D^{2}} {f_{1}}\bigl({V^{*}}\bigr){q_{0}} &= \frac{{{\partial ^{2}}{f_{1}}}}{{\partial {V^{2}}}} + \frac{2r}{C_{M}}\frac{{{\partial ^{2}}{f_{1}}}}{{\partial V\partial w}}\frac{{\partial {f_{2}}}}{{\partial V}} + \frac{r^{2}}{C_{M}^{2}}\frac{{{\partial ^{2}}{f_{1}}}}{{\partial {w^{2}}}}{ \biggl( {\frac{{\partial {f_{2}}}}{{\partial V}}} \biggr)^{2}}\\ & \quad{} + \frac{2}{C_{M}}\partial _{Va}^{f_{1}^{T}}\tau \partial _{V}^{{f_{3}}} + \frac{1}{C_{M}^{2}}\partial _{V}^{f_{3}^{T}}\tau \partial _{aa}^{{f_{1}}}\tau \partial _{V}^{{f_{3}}}, \end{aligned} \\& \frac{1}{{{q^{2}_{01}}}}q_{0}^{T}{D^{2}} {f_{2}}\bigl({V^{*}}\bigr){q_{0}} = \frac{{{\partial ^{2}}{f_{2}}}}{{\partial {V^{2}}}} + \frac{2C_{M}^{2}}{r^{2}}\frac{{dr}}{{dV}}\frac{{\partial {f_{2}}}}{{\partial V}}, \\& \frac{1}{{{q^{2}_{01}}}}q_{0}^{T}{D^{2}} {f_{3}}\bigl({V^{*}}\bigr){q_{0}}=\partial _{VV}^{{f_{3}}} + 2C_{M}^{2}{\tau ^{ - 2}}\partial _{V}^{\tau }\tau \partial _{V}^{{f_{3}}}, \end{aligned}$$

where $\partial _{aa}^{{f_{1}}} = \operatorname{diag}(\partial _{{a_{3}}{a_{3}}}^{{f_{1}}},\ldots,\partial _{{a_{N}}{a_{N}}}^{{f_{1}}})$. Consequently,

$$\begin{aligned} \frac{1}{{{p_{11}}q_{01}^{2}}}{\alpha _{2}} &= \frac{{{\partial ^{2}}{f_{1}}}}{{\partial {V^{2}}}} + \frac{2r}{C_{M}}\frac{{{\partial ^{2}}{f_{1}}}}{{\partial V\partial w}}\frac{{\partial {f_{2}}}}{{\partial V}} + \frac{r^{2}}{C_{M}^{2}} \frac{{{\partial ^{2}}{f_{1}}}}{{\partial {w^{2}}}}{ \biggl( {\frac{{\partial {f_{2}}}}{{\partial V}}} \biggr)^{2}} + \frac{2}{C_{M}}\partial _{Va}^{f_{1}^{T}}\tau \partial _{V}^{{f_{3}}}\\ &\quad{} +\frac{1}{C_{M}^{2}} \partial _{V}^{f_{3}^{T}}\tau \partial _{aa}^{{f_{1}}}\tau \partial _{V}^{{f_{3}}}+ \frac{r}{C_{M}}\frac{{\partial {f_{1}}}}{{\partial w}}\frac{{{\partial ^{2}}{f_{2}}}}{{\partial {V^{2}}}} + \frac{2C_{M}}{r} \frac{{\partial {f_{1}}}}{{\partial w}}\frac{{dr}}{{dV}}\frac{{\partial {f_{2}}}}{{\partial V}}\\ & \quad{} +\frac{1}{C_{M}} \partial _{a}^{f_{1}^{T}}\tau \partial _{uu}^{{f_{3}}} + 2C_{M}\partial _{a}^{f_{1}^{T}}{\tau ^{ - 1}}\partial _{V}^{\tau } \tau \partial _{V}^{{f_{3}}}. \end{aligned}$$

Recall that all of these derivatives are calculated at $V^{*}$. It follows from (8) that

$$\begin{aligned}& \begin{aligned} { {\frac{{{\partial ^{2}}{f_{2}}}}{{\partial {V^{2}}}}} \bigg| _{{E^{*}}}} &= C_{M}{r^{ - 1}}\bigl(V^{*}\bigr) \biggl( {{{ { \frac{{{d^{2}}}}{{d{V^{2}}}}{w_{\infty }}(V)} \bigg| }_{{V^{*}}}}} \biggr)\\ & \quad{} -2 C_{M}^{2}{r^{ - 2}} \biggl( {{{ { \frac{d}{{dV}}r(V)} \bigg| }_{{V^{*}}}}} \biggr) \biggl( {{{ { \frac{d}{{dV}}{w_{\infty }}(V)} \bigg| }_{{V^{*}}}}} \biggr), \end{aligned} \\& { {\partial _{VV}^{{f_{3}}}} \big| _{{E^{*}}}} = C_{M}{\tau ^{ - 1}}\bigl(V^{*}\bigr){ {\partial _{VV}^{{a_{\infty }}}} \big| _{{V^{*}}}} -2C_{M}^{2} {\tau ^{ - 2}}\bigl(V^{*}\bigr)\partial _{V}^{\tau }{ {\partial _{V}^{{a_{\infty }}}} \big| _{{V^{*}}}}. \end{aligned}$$

At $V=V^{*}$, we have $\frac{{\partial {f_{2}}}}{{\partial V}} =C_{M} {r^{ - 1}} \frac{{d{w_{\infty }}}}{{dV}}$ and $\partial _{V}^{{f_{3}}} = C_{M}{\tau ^{ - 1}}\partial _{V}^{{a_{ \infty }}}$. Hence,

$$\begin{aligned} \frac{1}{{{p_{11}}q_{01}^{2}}}{\alpha _{2}} &= \frac{{{\partial ^{2}}{f_{1}}}}{{\partial {V^{2}}}} + 2 \frac{{{\partial ^{2}}{f_{1}}}}{{\partial V\partial w}}\frac{{d{w_{\infty }}}}{{dV}} + \frac{{{\partial ^{2}}{f_{1}}}}{{\partial {w^{2}}}}{ \biggl( { \frac{{d{w_{\infty }}}}{{dV}}} \biggr)^{2}}\\ &\quad {} + \frac{{\partial {f_{1}}}}{{\partial w}}\frac{{{d^{2}}{w_{\infty }}}}{{d{V^{2}}}} + 2\partial _{Va}^{f_{1}^{T}}\partial _{V}^{{a_{\infty }}} + \partial _{V}^{a_{\infty }^{T}}\partial _{aa}^{{f_{1}}}\partial _{V}^{{a_{\infty }}} + \partial _{a}^{f_{1}^{T}}\partial _{VV}^{{a_{\infty }}}\\ &={ {\frac{d^{2}}{{dV^{2}}}{I_{\infty }}(V)} \bigg| _{{V^{*}}}}. \end{aligned}$$

Thus, $\alpha _{2}=0$ if and only if

$$ { {\frac{d^{2}}{{dV^{2}}}{I_{\infty }}(V)} \bigg| _{{V^{*}}}}=0. $$

Consequently, from (19), we have $A{h_{20}} = -G(q_{0},q_{0})$, which has infinite solutions. This system is consistent due to the Fredholm solvability condition [26]. Hence, $h_{20}$ can be chosen such that $\beta _{2}\ne 0$ in (18). This completes the proof. □

## Existence of bifurcations

Theorems 3.1 and 3.2 imply three bifurcations: BT, CP and BTC which are characterised by equations (5)–(7) and (20). In the following we discuss the solution of these equations. Recall that equation (5) relates the equilibrium point voltage value $V^{*}$ to $I_{app}$ and the other parameters.

Rearranging (6), we obtain

21 $$ -g_{M} X_{1}\bigl(V^{*}\bigr) + X_{2}\bigl(V^{*}\bigr)=g_{L}, $$

where

$$\begin{aligned}& X_{1}\bigl(V^{*}\bigr)={w^{*}} + \biggl( {{{ { \frac{d}{{dV}}{w_{\infty }}(V)} \bigg| }_{{V^{*}}}}} \biggr) \bigl( {{V^{*}} - V_{K}} \bigr), \\& X_{2}\bigl(V^{*}\bigr)={ {\frac{d}{{dV}}{I_{ion,\infty }}(V)} \bigg| _{{V^{*}}}}. \end{aligned}$$

Similarly, (7) leads to

22 $$ -g_{M} Y_{1}\bigl(V^{*}\bigr) +Y_{2}\bigl(V^{*}\bigr)=-1, $$

where

$$\begin{aligned}& Y_{1}\bigl(V^{*}\bigr)=\frac{r(V^{*})}{C_{M}} \bigl( {{V^{*}} - V_{K}} \bigr) \biggl( {{{ {\frac{d}{{dV}}{w_{\infty }}(V)} \bigg| }_{{V^{*}}}}} \biggr), \\& Y_{2}\bigl(V^{*}\bigr)=\frac{1}{C_{M}}{ {\partial _{a}^{{I_{ion}}}} \bigg| _{{E^{*}}}}\tau \bigl(V^{*} \bigr){ {\partial _{V}^{{a_{\infty }}}} \bigg| _{{V^{*}}}}. \end{aligned}$$

It is easy to check that the second derivative of $I_{\infty }(V)$ is

$$\begin{aligned} { {\frac{{{d^{2}}}}{{d{V^{2}}}}{I_{\infty }}(V)} \bigg| _{{V^{*}}}} &= - {g_{M}} \biggl[ {2{{ {\frac{{d{w_{\infty }}}}{{dV}}} \bigg| }_{{V^{*}}}} + {{ { \frac{{{d^{2}}{w_{\infty }}}}{{d{V^{2}}}}} \bigg| }_{{V^{*}}}}\bigl({V^{*}} - V_{K} \bigr)} \biggr] \\ &\quad{} + { {\frac{{{d^{2}}}}{{d{V^{2}}}}{I_{ion,\infty }}(V)} \bigg| _{{V^{*}}}}. \end{aligned}$$

Thus, (20) holds when

23 $$ -g_{M} Z_{1}\bigl(V^{*} \bigr)+Z_{2}\bigl(V^{*}\bigr)=0. $$

### Bogdanov–Takens bifurcation

Suppose that there is $V^{*}$ that satisfies

$$ \bigl[g_{L}-X_{2}\bigl(V^{*}\bigr) \bigr]Y_{1}\bigl(V^{*}\bigr)+X_{1} \bigl(V^{*}\bigr) \bigl(Y_{2}\bigl(V^{*}\bigr)+1 \bigr)=0, $$

with at least one of $X_{1}(V^{*})$, $Y_{1}(V^{*})$ non-zero. Then there is an equilibrium $E^{*}=(V^{*},w^{*},\mathbf{a}^{*})$ that undergoes a Bogdanov–Takens bifurcation at $(I_{app},g_{M})=(I_{app}^{*},g_{M}^{*})$, where

24 $$ g_{M}^{*}= \frac{X_{2}(V^{*})-g_{L}}{X_{1}(V^{*})}= \frac{Y_{2}(V^{*})+1}{Y_{1}(V^{*})} $$

and $I_{app}^{*}$ is given by (5).

### Cusp bifurcation

Suppose that there is $V^{*}$ that satisfies

$$ \bigl[g_{L}-X_{2}\bigl(V^{*}\bigr) \bigr]Z_{1}\bigl(V^{*}\bigr)+X_{1} \bigl(V^{*}\bigr)Z_{2}\bigl(V^{*}\bigr)=0 $$

with at least one of $X_{1}(V^{*})$, $Z_{1}(V^{*})$ non-zero. Then there is an equilibrium $E^{*}=(V^{*},w^{*},\mathbf{a}^{*})$ that undergoes a cusp bifurcation at $(I_{app},g_{M})=(I_{app}^{*},g_{M}^{*})$, where

25 $$ g_{M}^{*}=\frac{X_{2}(V^{*})-g_{L}}{X_{1}(V^{*})}= \frac{Z_{2}(V^{*})}{Z_{1}(V^{*})} $$

and $I_{app}^{*}$ is given by (5).

### Bogdanov–Takens–cusp bifurcation

Suppose that there is $V^{*}$ that satisfies

$$ \bigl[Y_{2}\bigl(V^{*}\bigr)+1\bigr]Z_{1} \bigl(V^{*}\bigr)-Y_{1}\bigl(V^{*} \bigr)Z_{2}\bigl(V^{*}\bigr)=0 $$

with at least one of $Y_{1}(V^{*})$, $Z_{1}(V^{*})$ non-zero. Then there is an equilibrium $E^{*}=(V^{*},w^{*},\mathbf{a}^{*})$ that undergoes a BTC bifurcation at $(I_{app},g_{M},g_{L})=(I_{app}^{*},g_{M}^{*},g_{L}^{*})$, where

$$\begin{aligned}& g_{M}^{*}=\frac{Y_{2}(V^{*})+1}{Y_{1}(V^{*})}= \frac{Z_{2}(V^{*})}{Z_{1}(V^{*})}, \\& g_{L}^{*}=X_{2}\bigl(V^{*} \bigr)-g_{M}^{*}X_{1}\bigl(V^{*}\bigr) \end{aligned}$$

and $I_{app}^{*}$ is given by (5).

### Remark 4.1

We have explicitly included the leak current in our formulation. The leak current is not necessary for the occurrence of the BT and CP bifurcations. If $g_{L}=0$, then equation (21) becomes

$$ -g_{M} X_{1}\bigl(V^{*}\bigr) + X_{2} \bigl(V^{*}\bigr)=0 $$

and the solution will go through as above. However, for the BTC bifurcation to occur, we must have another parameter to vary. We have shown that this third parameter can be the leak conductance $g_{L}$. Solving the equations in a different way shows that the capacitance $C_{M}$ could also be used.

### Implications

In the previous section we gave conditions which guarantee that a BT bifurcation can be induced by the variation of two parameters found in our general model: the applied current $I_{app}$ and the maximal conductance of the M-current $g_{M}$. Further, if the conditions are met, we gave explicit expressions for the bifurcation point in terms of $g_{M}$ and $I_{app}$. Near this bifurcation point the behaviour of the system will be described by the unfolding of the normal form (17) in terms of two parameters. The normal form and unfolding were first studied by [29, 30]. The details can also be found in [25, 27]. A key point for our work is that emanating out of the BT point are three codimension one bifurcation curves: Hopf bifurcation, saddle homoclinic bifurcation and saddle node (fold) of equilibria. A periodic orbit exists between the Hopf and homoclinic bifurcation curves, the stability of which depends on the sign of the coefficients $\alpha _{2}$, $\beta _{2}$ in (17). Thus the emergence of periodic solutions via a Hopf bifurcation can be linked to the presence of the BT point.

In the previous section, we also gave conditions which guarantee that a BTC bifurcation can be induced by $I_{app}$, $g_{M}$ and the conductance of the leak current $g_{L}$. The normal form and unfolding for the case considered in Theorem 3.2 was first studied in [31]; see also [21, 26]. There are various possibilities for the bifurcations in the unfolding which are determined by the higher order terms in the normal form. The key results for our analysis are that in the three-dimensional parameter space there are two curves of cusp bifurcations and two curves of BT bifurcations with a surface of Hopf bifurcation starting at one BT curve and ending at the other. Near one BT bifurcation the Hopf bifurcation is supercritical (produces an asymptotically stable periodic orbit), while at the other it is subcritical. There is a saddle-node (fold) of limit cycles bifurcation associated with the change in criticality of the Hopf bifurcation. Fixing the value of one parameter (such as the leak conductance $g_{L}$) amounts to taking a two-dimensional slice in the three-dimensional parameter space. Thus, in general one should expect to see some subset of the bifurcations we have just described.

## Numerical examples

In this section, we implement three examples with different ranges of $g_{M}$ corresponding to the range between the BT and cusp points. We apply our theoretical results and compare them with computations carried out with MATCONT [32]. We also construct bifurcation diagrams using MATCONT to explain the possible behaviour of each example. The labels used in these bifurcation diagrams are summarised in Table 1. Furthermore, we use the numerical solution of the model in each example to measure the frequency-current ($\mathrm{F}/\mathrm{I}$) curves which illustrate the neuronal excitability class.

**Table 1:** Labels used to mark bifurcation points in one- and two-parameter bifurcation diagrams

Label	Bifurcation
LP	limit point (fold/saddle-node) of equilibria
red/black H	super/subcritical Andronov–Hopf
LPC	limit point (fold) of cycles
BT	Bogdanov–Takens
CP	cusp
GH	generalised Hopf (Bautin)

### Example 1

In [20], Wang and Buzśaki proposed a model to study the fast neuronal oscillations in the neocortex and hippocampus during behavioural arousal. The model is based on an inhibitory basket cell in rat hippocampus. The model with the inclusion of the *M*-current can be written as

26 $$\begin{aligned} \begin{gathered} \begin{aligned} C_{m} \frac{{dV}}{{dt}} &= {I_{app}} - {g_{L}}(V - {V_{L}}) - {g_{M}}w(V - {V_{K}}) - {g_{Na}}m_{\infty }^{3} ( V )h(V - {V_{Na}}) \\ &\quad{} - {g_{K}} {n^{4}}(V - {V_{K}}), \end{aligned} \\ \frac{{dw}}{{dt}} = \frac{1}{{{\tau _{w}}(V)}} \bigl( {{w_{\infty }}(V) - w} \bigr), \\ \frac{{d\sigma }}{{dt}} = \frac{\phi }{{{\tau _{\sigma }}(V)}} \bigl( {{{ \sigma }}_{\infty }}(V) - \sigma \bigr),\quad \sigma \in \{h,n\}, \end{gathered} \end{aligned}$$

supplemented by the dynamics for the gating variables *h* and *n* as in (1). Parameter values and other details of the model are given in the Appendix.

**Figure 1:**
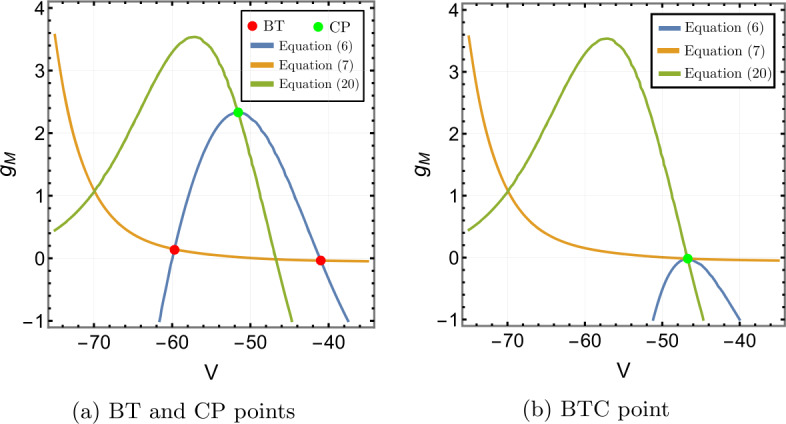
Existence of codimension two and three bifurcation points in Wang–Buzśaki model (26) with the parameter values given in Table 3. (**a**) The conditions given by equations (6), (7) and (20) are plotted in the *V*, $g_{M}$ space. The two intersection points (red dots) of the conditions in Theorem 3.1 show that there are two BT points in the model. The one intersection point (green dot) of the conditions in Theorem 3.2 show the existence of one cusp point; (**b**) The three conditions are plotted when the leak conductance is increased to $g_{L}=0.7507$. The intersection point (green dot) corresponds to the BTC point

Figure 1 shows the contour plot of equations (6), (7) and (20). In Fig. 1a, there are two intersections of equations (6) and (7) at $g_{M}=-0.0368$ and $g_{M}=0.1455$. Consequently, there are two BT points: $(V^{*},I_{app}^{*},g_{M}^{*})=(-40.9926,-6.7925,-0.0368)$ and $(-59.6978,0.2000,0.1455)$. The bio-physically permissible point is the latter one where $g_{M}>0$. Moreover, there is one intersection of (7) and (20) implying the cusp point is $(\widehat{V},\widehat{I}_{app},\widehat{g}_{M})=(-51.5531,1.2382,2.3316)$, see Fig. 1a.

The analysis of Sect. 4 shows that of the three curves, only the one defined by equation (6) depends on $g_{L}$. Further, the representation (21) of this equation and the properties of the M-current show that increasing $g_{L}$ will move this curve downward. Given the shape of the curves in Fig. 1a, it is clear that increasing $g_{L}$ will move the BT and CP points closer together, and for sufficiently large $g_{L}$ we should obtain a single intersection point of all three curves corresponding to a BTC point. Figure 1b confirms that when we increase $g_{L}$ to 0.7507, we find the (approximate) BTC point $(-46.6416,7.75907,-0.0166046)$.

We use the MATLAB numerical continuation package MATCONT [32] to verify the theoretical results and supplement them with numerical bifurcation diagrams. From MATCONT, we find two BT points $(V^{*},I_{app}^{*},g_{M}^{*})=(-59.698,0.2,0.146)$ and $(-40.992,-6.792, -0.036)$ (we omit this point) and one cusp point $(\widehat{V},\widehat{I}_{app},\widehat{g}_{M})=(-51.553,1.238,2.332)$ with parameter values in Table 3. This is consistent with our results in Fig. 1.

**Figure 2:**
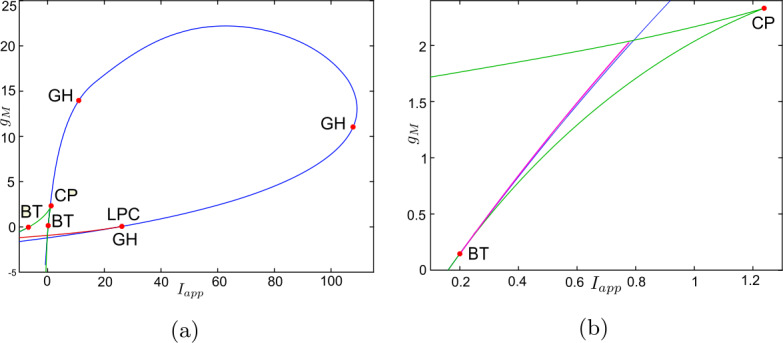
Bifurcation diagram in the $I_{app}$, $g_{M}$ parameter space for Wang–Buzśaki model (26). Green curves are limit point (fold/saddle-node) bifurcations of equilibria, blue are Andronov–Hopf bifurcations, magenta are homoclinic bifurcations and red are limit point (fold) bifurcations of limit cycles. Codimension two bifurcation point labels are described in Table 1

Now, we discuss the switch in the model neuronal excitability class as $g_{M}$ increases. We plot a bifurcation diagram in the $I_{app}$, $g_{M}$ parameter space for Wang–Buzśaki model (26) in Fig. 2. As expected from the normal form analysis, there is a curve of homoclinic bifurcations, a curve of Hopf bifurcation and a curve of saddle-node of equilibria emanating from the BT point. The Hopf is subcritical and thus an unstable periodic orbit exists for any parameters between the homoclinic and Hopf curves. See Fig. 2b. These curves are associated with the transition in the neuronal excitability class and show three cases.

**Figure 3:**
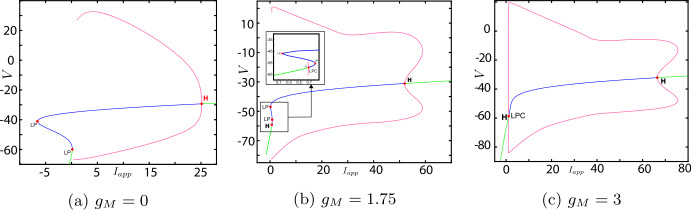
One-parameter bifurcation diagrams for Wang–Buzśaki model (26), showing the change in bifurcation structure as $g_{M}$ is varied. (**a**) $g_{M}< g_{M}^{*}$ (the value at the BT point); (**b**) $g_{M}^{*}< g_{M}<\widehat{g}_{M}$; (**c**) $g_{M}>\widehat{g}_{M}$ (the value at the CP point). Green/blue curves show stable/unstable equilibria. Pink curves show maxima/minima of periodic orbits. Codimension one bifurcation point labels are described in Table 1

**Figure 4:**
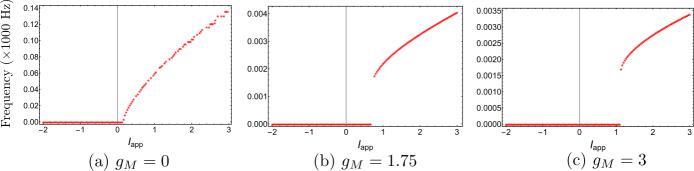
$\mathrm{F}/\mathrm{I}$ curves of Wang–Buzśaki model (26) corresponding to Fig. 3. (**a**) $g_{M}< g_{M}^{*}$ (the value at the BT point); (**b**) $g_{M}^{*}< g_{M}<\widehat{g}_{M}$; (**c**) $g_{M}>\widehat{g}_{M}$ (the value at the CP point)

**Figure 5:**
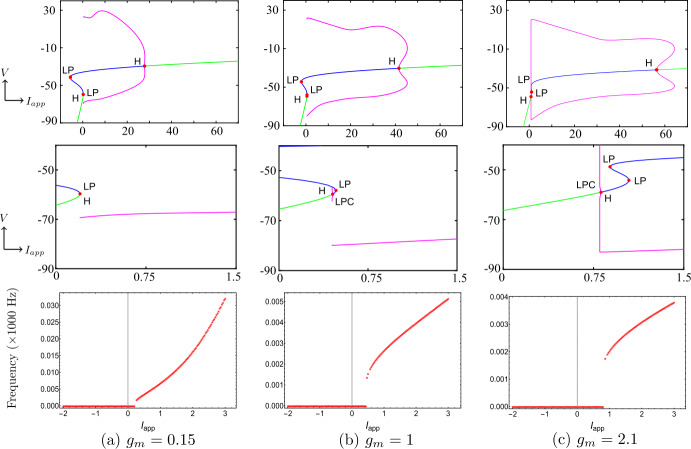
Top row/middle row: Details of the change in the bifurcation structure of Wang–Buzśaki model (26) when $g_{M}$ is varied between the BT point and the cusp point. Green/blue curves show stable/unstable equilibria. Pink curves show maxima/minima of periodic orbits. Codimension one bifurcation point labels are described in Table 1. Bottom row: corresponding $\mathrm{F}/\mathrm{I}$ curves

- $g_{M}< g_{M}^{*}$: In Fig. 3a, when $g_{M}=0$ and $I_{app}<0.16$, there exists a stable equilibrium point that determines the resting state and two unstable equilibria. As the applied current increases, the stable and one unstable fixed points collide in a saddle-node bifurcation point (“LP”). Consequently, a limit cycle is born simultaneously and emanates from the LP, that is, the limit cycle is created via a saddle-node on invariant circle bifurcation (“SNIC”). As expected, the oscillations on the limit cycle appear with arbitrarily slow frequency (see Fig. 4a), indicating Class-I excitability [5, 23].
- $g_{M}>\widehat{g}_{M}$: For large enough $g_{M}$, a different sequence of bifurcations is observed. In Fig. 3c, when $g_{M}=3$, at $I_{app}=1$, a limit point bifurcation of cycles “LPC” occurs giving rise to one unstable and one stable periodic orbit. Then, at $I_{app}=1.1416$, the unstable periodic orbit disappears in a subcritical Hopf bifurcation (subHopf) of the lone equilibrium point, destabilising it. Consequently, firing with a positive frequency appears via LPC, and hence neuronal excitability Class-II occurs [5, 23]. See Figs. 3c–4c;
- $g_{M}^{*}< g_{M}<\widehat{g}_{M}$: In this case, both subHopf and LP exist. The stable equilibrium point disappears by subHopf, and the LP occurs when two unstable equilibria collide. The model dynamics exhibits two different patterns, which are only distinguished by the bifurcations of the unstable periodic orbit(s). (i) When $g_{M}^{*}< g_{M}<2.1$, see Figs. 5a–5c and in Fig. 3b, an unstable limit cycle is created via a homoclinic bifurcation (the magenta curve in Fig. 2b) and disappears in the subHopf. In this case, the stable limit cycle appears via an LPC with a different unstable limit cycle which disappears via homoclinic orbit bifurcation (not shown in Fig. 2b). (ii) When $2.1\lesssim g_{M}<\widehat{g}_{M}$, the sequence of bifurcations is very similar to that for $g_{M}>\widehat{g}_{M}$. An LPC bifurcation creates both unstable and stable periodic orbits. The former is lost in the subHopf, see Fig. 5c. For all $g_{M}\in (g_{M}^{*},\widehat{g}_{M})$, there is a region of bistability between a stable limit cycle and a stable equilibrium point, between the LPC and subHopf bifurcations. Consequently, when $g_{M}^{*}< g_{M}<\widehat{g}_{M}$, a neuronal excitability Class-II occurs [5, 23].

Therefore, the model neuronal excitability type switches from Class-I to Class-II when the conductance of the M-current $g_{M}$ passes through the BT point.

Now let us consider the effect of the leak conductance $g_{L}$. As shown above, increasing $g_{L}$ monotonically decreases the $g_{M}$ value at the bio-physically permissible BT point. This means that the range of values of $g_{M}$ where the model has Class-I excitability will be decreased. Equivalently, smaller changes of $g_{M}$ are needed to switch the model from Class I to Class II. If $g_{L}$ is increased enough, then $g_{M}^{*}$ may become negative, in which case the model will exhibit Class-II excitability regardless of the value of $g_{M}$.

### Example 2

In [33], Stiefel et al. proposed a single-compartmental neuron model that included biophysically realistic mechanisms for neuronal spiking based on Hodgkin and Huxley ionic currents. The single-compartment Stiefel model can be written as follows:

27 $$\begin{aligned} \begin{gathered} \begin{aligned} C_{m} \frac{{dV}}{{dt}}&= {I_{app}} - {g_{L}}(V - {V_{L}}) - {g_{M}}w(V - {V_{K}}) - {g_{Na}}m_{\infty }^{3} ( V )h(V - {V_{Na}}) \\ &\quad{} - {g_{K}} {n^{4}}(V - {V_{K}}), \end{aligned} \\ \frac{{d\sigma }}{{dt}} = \frac{\phi _{\sigma } }{{{\tau _{\sigma }}(V)}} \bigl( {{{\sigma }}_{\infty }}(V) - \sigma \bigr),\quad \sigma \in \{w,h,n\}. \end{gathered} \end{aligned}$$

Parameter values and other details can be found in the Appendix.

**Figure 6:**
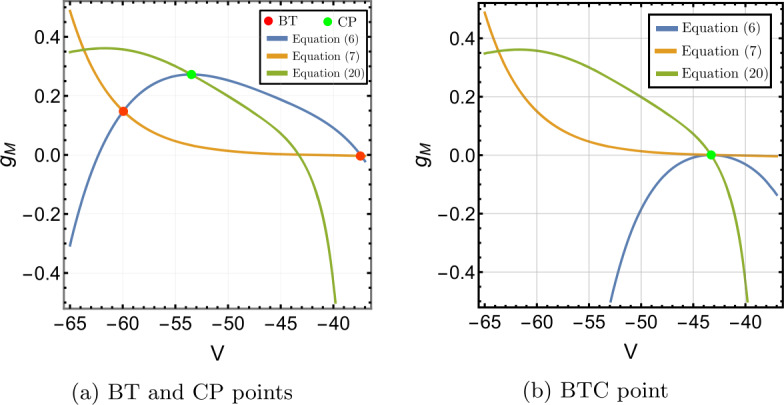
Existence of codimension two and three bifurcation points in Stiefel model (27) with the parameter values given in Table 4. (**a**) The conditions given by equations (6), (7) and (20) are plotted in the *V*, $g_{M}$ space. The two intersection points (red dots) of the conditions in Theorem 3.1 show that there are two BT points in the model. The one intersection point (green dot) of the conditions in Theorem 3.2 shows the existence of one cusp point; (**b**) The three conditions are plotted when the leak conductance is increased to $g_{L}=0.3785$. The intersection point (green dot) corresponds to the BTC point

**Figure 7:**
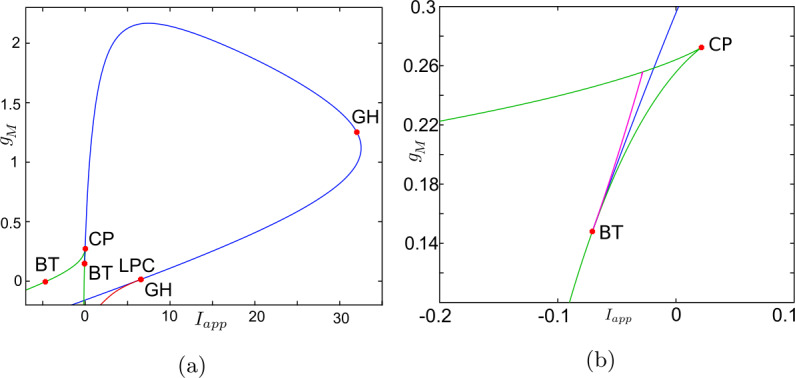
Bifurcation diagram in the $I_{app}$, $g_{M}$ parameter space for Stiefel model (27). Green curves are limit point (fold/saddle-node) bifurcations of equilibria, blue are Andronov–Hopf bifurcations, magenta are homoclinic bifurcations and red are limit point (fold) bifurcations of limit cycles (LPC). Codimension two bifurcation point labels are described in Table 1

**Figure 8:**
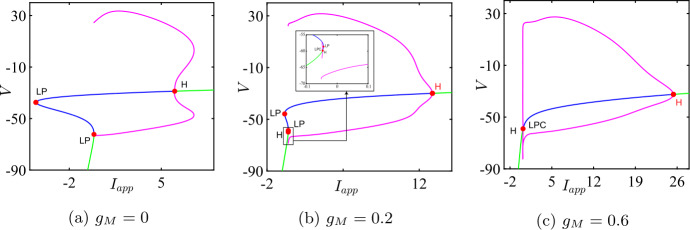
One-parameter bifurcation diagrams for Stiefel model (27), showing the change in the bifurcation structure as $g_{M}$ is varied. (**a**) $g_{M}< g_{M}^{*}$ (the value at the BT point); (**b**) $g_{M}^{*}< g_{M}<\widehat{g}_{M}$; (**c**) $g_{M}>\widehat{g}_{M}$ (the value at the CP point). Green/blue curves show stable/unstable equilibria. Pink curves show maxima/minima of periodic orbits. Codimension one bifurcation point labels are described in Table 1

**Figure 9:**
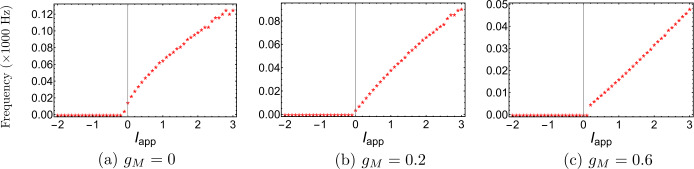
$\mathrm{F}/\mathrm{I}$ curves of Stiefel model (27) corresponding to Fig. 8. (**a**) $g_{M}< g_{M}^{*}$ (the value at the BT point); (**b**) $g_{M}^{*}< g_{M}<\widehat{g}_{M}$; (**c**) $g_{M}>\widehat{g}_{M}$ (the value at the CP point)

Solving equations (6), (7) and (20) leads to the BT point $(V^{*},I_{app}^{*},g_{M}^{*})=(-59.9344, -0.0707,0.1482)$ and the cusp point $(\widehat{V},\widehat{I}_{app},\widehat{g}_{M})=(-53.4754,0.0216,0.2724)$, see Figs. 6a. A second BT point occurs for $g_{M}<0$. These results are consistent with those found in MATCONT. Applying the analysis of Sect. 4 to this model also shows that increasing $g_{L}$ should lead to a BTC point. This is confirmed in Fig. 6b. We find the BTC point $(-43.1385,2.9461,0.0008)$ when we increase $g_{L}$ to 0.3785. As in the previous example, the neuronal excitability type switches from Class I to II as the conductances of the M-current increase, Class I when $g_{M}< g_{M}^{*}$ and Class II otherwise, see Figs. 7, 8 and 9. Although the range $(g_{M}^{*},\widehat{g}_{M})$ is much smaller than for Example 1, model (27) exhibits a similar behaviour in this range, see Fig. 5.

### Example 3

The reduced Traub–Miles (RTM) model is a substantial simplification of a model of a pyramidal excitatory cell in rat hippocampus due to Traub and Miles [34]. The RTM model with the M-current can be written as [35]

28 $$\begin{aligned}& \begin{aligned}[b] C_{m} \frac{{dV}}{{dt}} &= {I_{app}} - {g_{L}}(V - {V_{L}}) - {g_{M}}w(V - {V_{K}}) - {g_{Na}}m^{3} h(V - {V_{Na}}) \\ &\quad{} - {g_{K}} {n^{4}}(V - {V_{K}}), \end{aligned} \\& \frac{{d\sigma }}{{dt}} = \frac{1}{{{\tau _{\sigma }}(V)}} \bigl( {{{ \sigma }}_{\infty }}(V) - \sigma \bigr),\quad \sigma \in \{w,h,n,m\}. \end{aligned}$$

Parameter values and other details are given in the Appendix.

**Figure 10:**
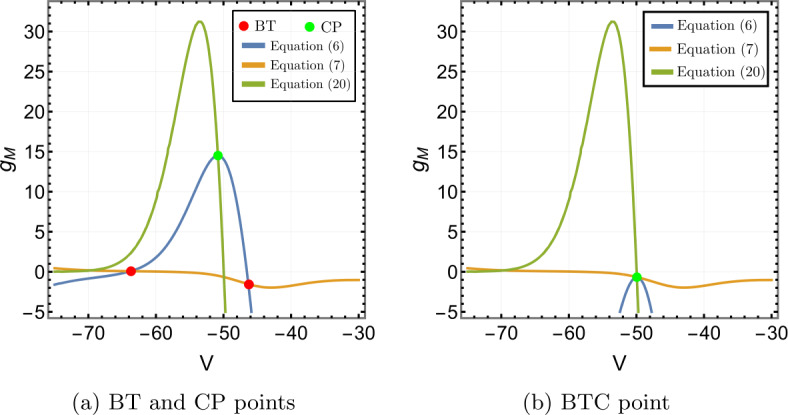
Existence of codimension two and three bifurcation points in Wang–Buzśaki model (28) with the parameter values given in Table 5. (**a**) The conditions given by equations (6), (7) and (20) are plotted in the *V*, $g_{M}$ space. The two intersection points (red dots) of the conditions in Theorem 3.1 show that there are two BT points in the model. The one intersection point (green dot) of the conditions in Theorem 3.2 shows the existence of one cusp point; (**b**) The three conditions are plotted when the leak conductance is increased to $g_{L}=13.79$. The intersection point (green dot) corresponds to the BTC point

**Figure 11:**
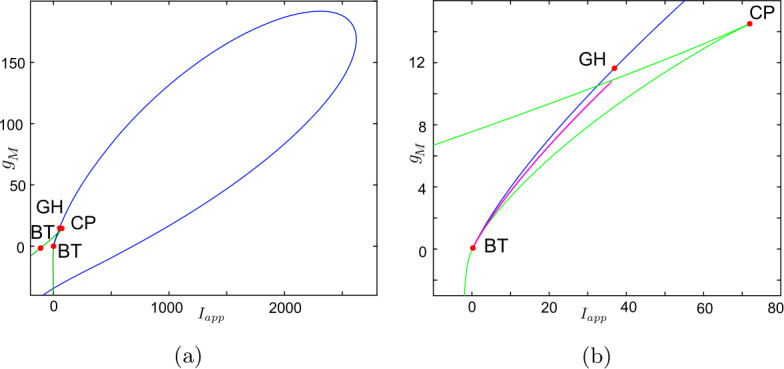
Bifurcation diagram in the $I_{app}$, $g_{M}$ parameter space for RTM model (28). Green curves are limit point (fold/saddle-node) bifurcations of equilibria, blue are Andronov–Hopf bifurcations, magenta are homoclinic bifurcations and red are limit point (fold) bifurcations of limit cycles (LPC). Codimension two bifurcation point labels are described in Table 1

**Figure 12:**
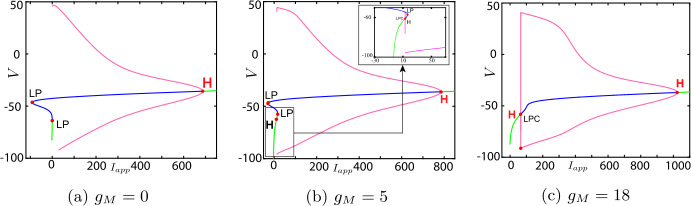
One-parameter bifurcation diagrams for RTM model (28), showing the change in the bifurcation structure as $g_{M}$ is varied. (**a**) $g_{M}< g_{M}^{*}$ (the value at the BT point); (**b**) $g_{M}^{*}< g_{M}<\widehat{g}_{M}$; (**c**) $g_{M}>\widehat{g}_{M}$ (the value at the CP point). Green/blue curves show stable/unstable equilibria. Pink curves show maxima/minima of periodic orbits. Codimension one bifurcation point labels are described in Table 1

Both the analytical results and MATCONT give the bio-physically permissible BT point $(V^{*},I_{app}^{*},g_{M}^{*})=(-63.7386,0.2449,0.0659)$ and the cusp point $(\widehat{V},\widehat{I}_{app},\widehat{g}_{M})=(-50.8204, 71.9395,14.5123)$, see Fig. 10a. Applying the analysis of Sect. 4 again shows that increasing $g_{L}$ should lead to a BTC point. This is confirmed in Fig. 10b. When we increase $g_{L}$ to 13.79, the BT and CP points collide producing the BTC point $(-49.8762,166.25,-0.6745)$. In this example, we notice that the range $(g_{M}^{*},\widehat{g}_{M})$ is bigger than those in Example 1 and 2, but the transition in the neuronal excitability type is consistent with previous examples: Class I when $g_{M}< g_{M}^{*}$ and Class II otherwise, see Figs. 11 and 12.

## Implications for synchronisation

In Sect. 4 we showed that the M-current will give rise to a BT bifurcation in any conductance-based neural model, when certain conditions are met. In Sect. 5 we showed in three examples that these conditions are met and a BT bifurcation occurs. Further, we showed that this BT bifurcation induces a transition from Class-II to Class-I excitability in these models as the conductance of the M-current is decreased (as would be the case in the presence of acetylcholine). In this section we explore one implication of this transition. There are many studies in the literature describing the relationship between the synchronisation of coupled neurons and their neuronal excitability type, see, e.g. [8, 24]. The classic result is that the in-phase solution of a pair of weakly coupled Class-I oscillators model with synaptic coupling is stable when there are inhibitory coupling and unstable for excitatory coupling, while the anti-phase solution exhibits the opposite stability [24]. The synchronisation of Class-II oscillators is less clear, and other factors such as the synaptic time constants and firing frequency may affect these conclusions [24, 33]. By in-phase solution, we mean both oscillators reach their highest peak at the same time, whereas an anti-phase solution means one oscillator reaches its highest peak one half-period after the other oscillator.

To study the stability of phase-locked solutions and the correspondence with the neuronal excitability type as $g_{M}$ varies, we write two coupled neurons with synaptic coupling as follows:

29 $$\begin{aligned}& {C_{m}}\frac{{d{V_{i}}}}{{dt}} = {I_{app}} - {g_{L}}({V_{i}} - {V_{L}}) - {g_{M}}w({V_{i}} - {V_{K}}) -I_{ion}(V)- {g_{syn}} {s_{j}}({V_{i}} - {V_{syn}})),\\& \frac{{dw}}{{dt}} = \frac{{{1}}}{{{\tau _{w}}(V)}} \bigl( {{w_{\infty }}({V_{i}}) - w} \bigr), \\& \frac{{d{s_{i}}}}{{dt}} ={a_{{e_{0}}}} {a_{e}}(V) (1 - {s_{i}})-\frac{{ {s_{i}}}}{{{\tau _{s}}}} \end{aligned}$$

for $i,j=1,2$ such that $i\ne j$, where $I_{ion}$ are ionic currents in Examples 1–3. The synaptic coupling function and parameters are given in Table 2.

**Table 2:** Synaptic coupling function and parameters in (29)

	${a_{{e_{0}}}}$	${\tau _{s}}$	${a_{e}}(V)$	Reference
Example 1: $V_{syn}=0,-75$	6.25	5	${{{ ( {1 + \exp ( {\frac{{ - V}}{2}} )} )}^{ - 1}}} $	[36]
Example 2: $V_{syn}=0,-80$	4	8	${{{ ( {1 + \exp ( {\frac{{ - V}}{5}} )} )}^{ - 1}}} $	[1]
Example 3: $V_{syn}=0$	5	2	(1 + tanh(*V*/4))	[35]
Example 3: $V_{syn}=-80$	2	10	(1 + tanh(*V*/4))	[35]

**Figure 13:**
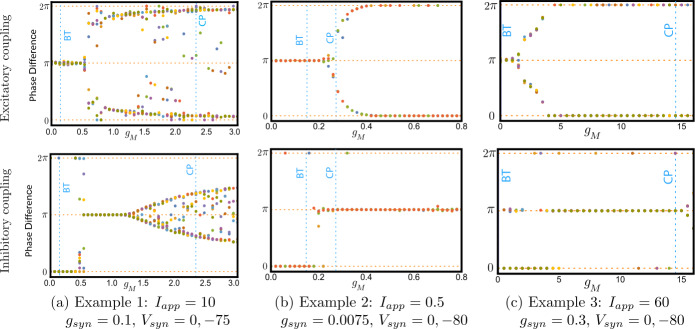
Bifurcation diagrams showing the change of synchronisation of two identical, synaptically coupled neurons as $g_{M}$ is varied. For all examples with $g_{M}< g_{M}^{*}$ (the BT point) excitatory coupling leads to phase-locking in anti-phase (phase difference *π*), while inhibitory coupling leads to in-phase (phase difference 0). In all cases $g_{M}$ has to be increased significantly past the *BT* value before the solution switches to in-phase for excitatory coupling and anti-phase for inhibitory coupling

To determine the stable phase-locked solution(s), first we solve (29) numerically with ten random initial conditions at each step of $g_{M}$, then we calculate the period of the oscillators ($T_{1}$ and $T_{2}$) in the numerical solution. Finally, we approximate the phase shift as

30 $$ \begin{aligned} \varphi = 2\pi \biggl( {\frac{\tau }{\mathcal{T}} - \biggl\lfloor { \frac{\tau }{\mathcal{T}}} \biggr\rfloor } \biggr), \end{aligned} $$

where $\lfloor \cdot \rfloor $ is the floor function, $\mathcal{T}=(T_{1}+T_{2})/2$ and *τ* is the argument shift satisfying $V_{1}(t)=V_{2}(t+\tau )$ for all *t*. Figure 13 shows bifurcation diagrams for (29) with excitatory and inhibitory synaptic coupling in Examples 1–3. For instance, for coupled Wang–Buzsaki model (Example 1), we notice in Fig. 13a that when $g_{M}< g_{M}^{*}$ (Class-I dynamics in (26)), the in-phase solution is unstable and the anti-phase solution is stable with excitatory coupling $V_{syn}=0$. The reverse is true for inhibitory coupling $V_{syn}=-75$. This is consistent with [24]. When there is an excitatory synaptic connection, as the M-current reaches $g_{M}\approx 0.5$, the anti-phase solution loses its stability and two stable out-of-phase solutions (neither in-phase nor anti-phase) appear. As the conductance of the M-current is increased any further, a stable in-phase solution appears. Hence, there is a transition from stable anti-phase solution to stable in-phase solution via stable out-of-phase solutions. The transition also occurs at $g_{M}\approx 0.5$ when there is the coupling is inhibitory. We observe a similar dynamical behaviour in Examples 2 and 3, see Fig. 13b–13c, although the transition is not as clear in all cases. Although the relationship of the transition point to the codimension two bifurcations varies with the different models, in all cases it occurs at some $g_{M}\in (g_{M}^{*},\widehat{g}_{M})$, that is, when the model has Class-II dynamics.

As indicated above, other factors may affect the synchronisation of neurons. We focus here on the firing frequency of the neuron. In [33] it was shown that increasing the firing frequency by increasing the applied current could switch the PRC of a model neuron with an M-current from Type-II to closer to Type-I. In [13], the authors reproduced this result for other neural models and studied how changing the firing frequency modulates the synchronisation properties induced by the M-current. They found that synchrony in excitatory networks of neurons with a Type I PRC (low $g_{M}$) was largely unaffected by frequency modulation, whereas networks of Type II PRC neurons (high $g_{M}$) synchronised much better at lower frequencies. In [7], the authors studied how the stability of in-phase and anti-phase phase-locked solutions in Wang–Buzśaki model (with no M-current) varied with firing frequency. At low frequencies with inhibitory coupling, they showed that both in-phase and anti-phase phase-locked solutions were stable. However, at higher frequencies only the in-phase solution was stable. In contrast, with excitatory coupling, they showed that the in-phase solution was unstable for both high and low frequencies. Recalling that the Wang–Buzśaki model is a Class-I oscillator, this latter result is consistent with that of [13].

**Figure 14:**
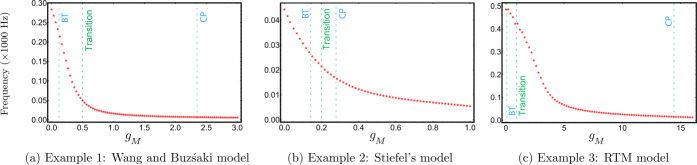
$\mathrm{F}/g_{M}$ curve of the models in Examples 1–3. For each model neuron, the applied current was fixed at a value which yielded stable periodic solutions for all $g_{M}$ in the given range, then the frequency of the periodic solution was plotted against $g_{M}$. The blue dashed lines show the $g_{M}$ values corresponding to the BT and CP points. The green dashed line shows the $g_{M}$ value where the change in synchronisation occurs for the coupled neurons in Fig. 13

To consider if firing frequency has an effect in our results, we determined the variation of firing frequency with the conductance of the M-current $g_{M}$ for our example models, see Fig. 14. In all cases the firing frequency decreases rapidly as $g_{M}$ increases. When the models are in the Class-I excitability regime (below the BT point), the frequency change does not affect the synchronisation properties. This is consistent with the results described above [13, 33], given that neurons with Class-I excitability typically have Type-I PRCs [24]. Recalling that the main switch in synchronisation behaviour in all cases occurs within the Class-II regime, we conclude that this switch is likely due to the decrease in the frequency as $g_{M}$ increases.

In summary, while the excitability class of the model changes exactly at the BT point, the synchronisation property of the models switches at $g_{M}$ value larger than the BT point, when the frequency of the intrinsic oscillations is small enough.

## Discussion

In this paper, we studied Bogdanov–Takens (BT) bifurcation in a general conductance-based neuron model with the inclusion of the M-current. We started by showing the existence of equilibrium points. Then we derived the necessary and sufficient conditions for the equilibrium point to become a BT point. A degenerate Bogdanov–Takens (BTC) point appears when BT and cusp points merge. To discuss the occurrence of such a point, we provided the condition for a cusp bifurcation. We then showed that the conditions for the BT and cusp bifurcation may be satisfied by varying the applied current and the maximal conductance of the M-current and that for the BTC point by additionally varying the conductance of the leak current.

As previously noted, our theoretical work was inspired by two recent papers. In [21] they show that the BTC point can occur in any conductance-based model in the parameter space of the applied current, leak conductance and capacitance. They use this to study the effect of the leak current on the excitability properties of models for single neurons and synchronisation properties for networks of neurons. In [22] they study a general conductance-based neural model. They show that if the model has an equilibrium point with a double zero eigenvalue for some parameter values, then it is a BT point. Further, they give conditions on the gating variables and time constants for a BT bifurcation to occur. They propose the BT normal form as a generic minimal model for a single neuron.

Numerically, we applied our analytical results to three examples and compared them with the computations of MATCONT, a numerical bifurcation analysis toolbox in Matlab. Furthermore, we constructed bifurcation diagrams using MATCONT to explain the possible behaviour of each example and discuss the switches in the neuronal excitability class with respect to the M-current $g_{M}$. As predicted by normal form theory [25, 27, 29, 30] in all examples a curve of homoclinic bifurcation, a curve of Hopf bifurcation and a curve of saddle-node of equilibria emanate from the BT point. These latter two curves particularly affect the neuronal excitability class. We found that a transition is determined by the BT point which occurs at $(g_{M},I_{app})=(g_{M}^{*},I_{app}^{*})$. The model is a Class-I oscillator when $g_{M}< g_{M}^{*}$ and Class-II when $g_{M}>g_{M}^{*}$. More precisely, when $g_{M}< g_{M}^{*}$ as $I_{app}$ is increased, oscillations with arbitrarily slow frequency appear via a saddle-node on invariant circle bifurcation, while when $g_{M}>g_{M}^{*}$ oscillations with a positive frequency appear via a fold bifurcation of cycles, followed by a subcritical Hopf bifurcation.

Using systems of two synaptically coupled cells, we explored how the change in excitability class with the variation of $g_{M}$ affects synchronisation in the example models. We found that while the excitability class of the model changes exactly at the BT point, the synchronisation property of all the models switches at $g_{M}$ value larger than the BT point. We attributed this change to the fact that the M-current also affects the frequency of the intrinsic oscillations and that the synchronisation of Class-II oscillators has been shown to be sensitive to intrinsic frequency. Thus the necessary condition for the switch of synchronisation, we observed, is that the system be Class-II and the frequency be sufficiently small.

We also considered the effect of the leak conductance $g_{L}$ showing that, in the examples we considered, increasing $g_{L}$ decreases the $g_{M}$ value of the BT point. This means that the range of values of $g_{M}$ where the model has Class-I excitability will be decreased. Equivalently, smaller changes of $g_{M}$ are needed to switch the model from Class I to Class II. If $g_{L}$ is increased enough, then $g_{M}^{*}$ may become negative, in which case the model will exhibit Class II excitability regardless of the value of $g_{M}$. Since the switch of synchronisation occurs at a higher value of $g_{M}$ than the BT point, this does not necessarily mean that the system will not exhibit changes in synchronisation associated with a change in $g_{M}$, it just means that smaller changes in $g_{M}$ are needed to switch the synchronisation property. We note that Prescott et al. [11, 12] represented the increase in membrane conductance due to background synaptic input using a leak current with a reversal potential near rest in a Morris–Lecar model with an M-current. The one-parameter bifurcation diagrams in [12] are consistent with what we have seen in our analysis.

Our analysis of the effect of $g_{L}$ on the BT point relies on understanding how the intersection points of two curves vary with $g_{L}$. Only one curve depends on $g_{L}$, and we can show in general (i.e. for any model) that the curve will move downward as $g_{L}$ increases. This effect depends on two aspects of the M-current: the reversal potential is a large negative value (since it is a potassium current) and the current is non-inactivating, see equation (24).

The implications of these results for the action of acetylcholine are as follows. If the neuron is of Class-II in the absence of acetylcholine (corresponding to high $g_{M}$), then the presence of acetylcholine may push the system past the BT bifurcation point and change the neural excitability type to Class-I. The expected synchronisation in the presence of sufficient acetylcholine is then clear: neurons with excitable coupling will likely desynchronise, while those with inhibitory coupling will synchronise. This is consistent with the changes to the PRCs induced by acetylcholine observed in [1]. Whether or not acetylcholine induces a change in synchronisation may depend on intrinsic firing frequencies of cells. Expanding on the idea of Prescott et al. [11, 12], an increase in membrane input conductance would make the system more sensitive to the effects of acetylcholine, so that switches of synchronisation could occur more easily.

These conclusions, of course, assume that the only effect of acetylcholine is to down-regulate the M-current. However, acetycholine has been observed to have other effects, including down-regulating an afterhyperpolarization current $I_{AHP}$ [3, 37] and the leak current [1]. As indicated above, our work indicates that decreasing $g_{L}$ will increase the value of $g_{M}^{*}$. Thus the simultaneous down-regulation of the leak and M-currents would cause the switch of excitability class at higher values of $g_{M}$. The net effect would be to increase the sensitivity of the model to acetylcholine. We leave the exploration of the effect of the $I_{AHP}$ current for future work.

The effect of acetylcholine, through the M-current, on the synchronisation of cells has been explored using numerical simulations and phase response curves [1, 7, 13, 33]. We have linked these effects to a particular bifurcation structure of conductance-based models with an M-current and given conditions for this to occur in any conductance-based model. This approach allows us to generalise previous results and to easily explore the effect of multiple parameters in these models.

## Data Availability

Not applicable.

## Appendix: Parameters, units and functions used in Sect. 5

- *Example **1**: Wang–Buzśaki model.* The infinity and $\tau _{\sigma }$, $\sigma \in \{m,h,n\}$, functions are
  $$\begin{aligned} &{m_{\infty }}(V) = \frac{{{\alpha _{m}}(V)}}{{{\alpha _{m}}(V) + {\beta _{m}}(V)}},\quad\quad {h_{\infty }}(V) = \frac{{{\alpha _{h}}(V)}}{{{\alpha _{h}}(V) + {\beta _{h}}(V)}},\\ &{n_{\infty }}(V) = \frac{{{\alpha _{n}}(V)}}{{{\alpha _{n}}(V) + {\beta _{n}}(V)}},\quad\quad {w_{\infty }}(V) = \frac{1}{{{e^{ - \frac{{V + 27}}{7}}} + 1}} ,\\ &{\tau _{w}}(V) = \frac{1}{0.003 (e^{\frac{V+63}{15}}+e^{\frac{-(V+63)}{15}} )},\quad\quad {\tau _{h}}(V) = \frac{1}{{{\alpha _{h}(V)} + {\beta _{h}(V)}}},\\ & {\tau _{n}}(V) = \frac{1}{{{\alpha _{n}(V)} + {\beta _{n}(V)}}}, \end{aligned}$$
  where the rate constants $\alpha _{\sigma }$ and $\beta _{\sigma }$ are:
  $$\begin{aligned} &{\alpha _{m}}(V) = - \frac{{0.1(V + 35)}}{{{e^{ - 0.1(V + 35)}} - 1}},\quad\quad {\alpha _{h}}(V) = 0.07{e^{ - \frac{{V + 58}}{{20}}}},\\ &{\alpha _{n}}(V) = - \frac{{0.01(V + 34)}}{{{e^{ - 0.1(V + 34)}} - 1}},\quad \quad {\beta _{n}}(V) = 0.125{e^{ - \frac{{V + 44}}{{80}}}},\\ &{\beta _{m}}(V) = 4{e^{ - \frac{{V + 60}}{{18}}}},\quad\quad {\beta _{h}}(V) = \frac{1}{{{e^{ - 0.1(V + 28)}} + 1}}. \end{aligned}$$
  Parameter values are listed in Table 3.
  **Table 3:** Parameter values for Example 1: Wang–Buzśaki model (26)
  Conductance (mS/cm^2^)	Reversal potential (mV)	Capacitance (*μ*F/cm)	Others
  $g_{L}=0.1$	$V_{L}=-65$	$C_{M}=1$	*ϕ* = 5
  $g_{Na}=35$	$V_{Na}=55$		
  $g_{K}=9$	$V_{K}=-90$
  **Table 4:** Parameter values for Example 2: Stiefel model
  Conductance (mS/cm^2^)	Reversal potential (mV)	Capacitance (*μ*F/cm)	Others
  $g_{L}=0.02$	$V_{L}=-60$	$C_{M}=1$	$\phi _{w}=1$
  $g_{Na}=24$	$V_{Na}=55$		$\phi _{h}=1$
  $g_{K}=3$	$V_{K}=-90$		$\phi _{n}=1$
  **Table 5:** Parameter values for Example 3: RTM model (28)
  Conductance (mS/cm^2^)	Reversal potential (mV)	Capacitance (*μ*F/cm)
  $g_{L}=0.1$	$V_{L}=-67$	$C_{M}=1$
  $g_{Na}=100$	$V_{Na}=50$	
  $g_{K}=80$	$V_{K}=-100$	
- *Example **2**: Stiefel model.* The functions are:
  $$\begin{aligned} &{m_{\infty }}(V) = \frac{1}{e^{\frac{-(V+30)}{9.5}}+1},\quad\quad {w_{\infty }}(V) = \frac{1}{e^{\frac{-(V+39)}{5}}+1},\\ & {h_{\infty }}(V) = \frac{1}{e^{\frac{V+53}{7}}+1},\quad\quad \tau _{w}(V)=75,\\ &{n_{\infty }}(V) = \frac{1}{e^{\frac{-(V+30)}{10}}+1},\quad\quad \tau _{h}(V)=0.37+ \frac{2.78}{e^{\frac{V+40.5}{6}}+1},\\ &\tau _{n}(V)=0.37+\frac{1.85}{e^{\frac{V+27}{15}}+1}. \end{aligned}$$
- *Example **3**: Reduced Traub–Miles model.* The infinity and $\tau _{\sigma }$, $\sigma \in \{m,h,n,w\}$, functions are:
  $$\begin{aligned} &{m_{\infty }}(V) = \frac{{{\alpha _{m}}(V)}}{{{\alpha _{m}}(V) + {\beta _{m}}(V)}},\quad\quad {h_{\infty }}(V) = \frac{{{\alpha _{h}}(V)}}{{{\alpha _{h}}(V) + {\beta _{h}}(V)}},\\ &{n_{\infty }}(V) = \frac{{{\alpha _{n}}(V)}}{{{\alpha _{n}}(V) + {\beta _{n}}(V)}},\quad\quad {w_{\infty }}(V) = \frac{1}{{{e^{\frac{{ - ( {V + 35} )}}{{10}}}} + 1}},\\ & {\tau _{w}}(V) = \frac{{400}}{{3.3{e^{\frac{{V + 35}}{{20}}}} + {e^{\frac{{ - (V + 35)}}{{20}}}}}},\quad\quad {\tau _{n}}(V) = \frac{1}{{{\alpha _{n}(V)} + {\beta _{n}(V)}}},\\ &{\tau _{m}}(V) = \frac{1}{{{\alpha _{m}(V)} + {\beta _{m}(V)}}},\quad\quad {\tau _{h}}(V) = \frac{1}{{{\alpha _{h}(V)} + {\beta _{h}(V)}}}, \end{aligned}$$
  where the rate constants $\alpha _{\sigma }$ and $\beta _{\sigma }$ are:
  $$\begin{aligned} &{\alpha _{m}}(V) = \frac{{0.32(V + 54)}}{{1 - {e^{ - \frac{{V + 54}}{4}}}}},\quad \quad {\alpha _{h}}(V) = 0.128{e^{ - \frac{{V + 50}}{{18}}}},\\ & {\alpha _{n}}(V) = \frac{{0.032(V + 52)}}{{1 - {e^{ - \frac{{V + 52}}{5}}}}},\quad \quad {\beta _{n}}(V) = 0.5{e^{ - \frac{{V + 5}}{{40}}}},\\ &{\beta _{m}}(V) = \frac{{0.28(V + 27)}}{{{e^{\frac{{V + 27}}{5}}} - 1}},\quad \quad {\beta _{h}}(V) = \frac{4}{{{e^{ - \frac{{V + 27}}{5}}} + 1}}. \end{aligned}$$
